# [Corrigendum] Jak3 is involved in CCR7‑dependent migration and invasion in metastatic squamous cell carcinoma of the head and neck

**DOI:** 10.3892/ol.2024.14712

**Published:** 2024-10-02

**Authors:** Zhongti Zhang, Fayu Liu, Zhenning Li, Dan Wang, Ruiwu Li, Changfu Sun

Oncol Lett 13: 3191–3197, 2017; DOI: 10.3892/ol.2017.5861

Following the publication of the above article, an interested reader drew to the authors’ attention that two pairs of data panels showing the results of Transwell migration and invasion assays shown in Fig. 3B on p. 3195 contained overlapping sections, such that data which were intended to show the results of differently performed experiments may have been derived from the same original source(s). Furthermore, upon assessing the data in this paper independently in the Editorial Office, a further pair of data panels in Fig. 3B were found to be affected in a similar way.

After having re-examined their original data, the authors have realized that they assembled certain of the data (namely, the migration assay experiments) incorrectly into this figure. The revised version of Fig. 3, including the correct data for the migration assay experiments in Fig. 3B (the upper row of data panels) is shown on the next page. Note that the errors made during the assembly of this figure did not affect the overall conclusions reported in the paper. All the authors agree with the publication of this corrigendum. The authors are grateful to the Editor of *Oncology Letters* for allowing them the opportunity to publish this, and also apologize to the readership for any inconvenience caused.

## Figures and Tables

**Figure 3.**
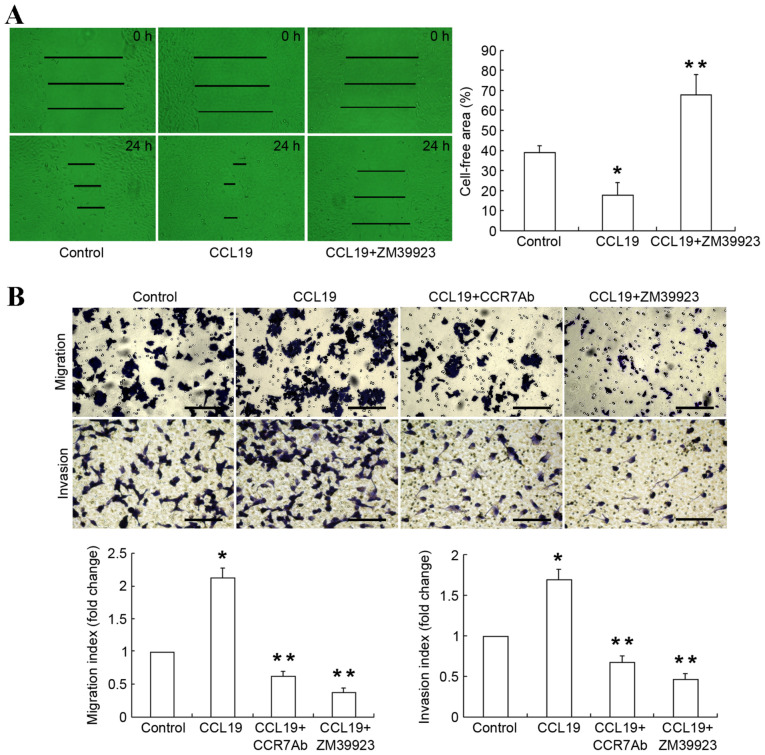
Jak3 activation enhances metastatic potential of SCCHN cells. (A) The cell migration rate was compared by wound healing assay. Wound closure was followed at 0 and 24 h subsequent to scratching the cell layer. (B) ZM39923 attenuated the cell migration and invasion induced by CCL19 application. Representative images and corresponding statistical data. Scale bars, 100 µm. Data are presented as the mean ± standard deviation (n=3, each group). *P<0.05 vs. the control group, **P<0.05 vs. group of CCL19 alone. SCCHN, squamous cell carcinoma of the head and neck; CCL19, chemokine ligand 19; CCR7, chemokine receptor 7..

